# An analysis of usability evaluation practices and contexts of use in wearable robotics

**DOI:** 10.1186/s12984-021-00963-8

**Published:** 2021-12-09

**Authors:** Jan Thomas Meyer, Roger Gassert, Olivier Lambercy

**Affiliations:** 1 Rehabilitation Engineering Laboratory, Department of Health Sciences and Technology, ETH Zurich, Zurich, Switzerland; 2 Future Health Technologies, Singapore-ETH Centre, Campus for Research Excellence And Technological Enterprise (CREATE), Singapore, Singapore

**Keywords:** Wearable robotics, Usability evaluation, User-centered design, Exoskeletons

## Abstract

**Background:**

User-centered design approaches have gained attention over the past decade, aiming to tackle the technology acceptance issues of wearable robotic devices to assist, support or augment human capabilities. While there is a consensus that usability is key to user-centered design, dedicated usability evaluation studies are scarce and clear evaluation guidelines are missing. However, the careful consideration and integration of user needs appears to be essential to successfully develop an effective, efficient, and satisfactory human-robot interaction. It is primarily the responsibility of the developer, to ensure that this users involvement takes place throughout the design process.

**Methods:**

Through an online survey for developers of wearable robotics, we wanted to understand how the design and evaluation in actual daily practice compares to what is reported in literature. With a total of 31 questions, we analyzed the most common wearable robotic device applications and their technology maturity, and how these influence usability evaluation practices.

**Results:**

A total of 158 responses from a heterogeneous population were collected and analyzed. The dataset representing contexts of use for augmentation (16.5%), assistance (38.0%), therapy (39.8%), as well as few other specific applications (5.7%), allowed for an insightful analysis of the influence of technology maturity on user involvement and usability evaluation. We identified functionality, ease of use, and performance as the most evaluated usability attributes and could specify which measures are used to assess them. Also, we could underline the frequent use of qualitative measures alongside the expected high prevalence of performance-metrics. In conclusion of the analysis, we derived evaluation recommendations to foster user-centered design and usability evaluation.

**Conclusion:**

This analysis might serve as state-of-the-art comparison and recommendation for usability studies in wearable robotics. We believe that by motivating for more balanced, comparable and user-oriented evaluation practices, we may support the wearable robotics field in tackling the technology acceptance limitations.

**Supplementary Information:**

The online version contains supplementary material available at 10.1186/s12984-021-00963-8.

## Background

Wearable robotic devices (WRD) to assist, support, or augment human functions and activities are gaining popularity and practicability. A great diversity from fully wearable and soft, to stationary and rigid devices has been developed over the past decades, showing promising ranges of functionalities [1–3]. Nonetheless, the number of WRD in daily use is still very low due to missing availability on the market, as well as technology acceptance limitations [1, 4, 5]. More specifically, the usability—defined as “extent to which a system, product or service can be used by specified users to achieve specified goals with effectiveness, efficiency, and satisfaction in a specified context of use” [6]—appears to be a key factor limiting the market translation and technology acceptance of WRD. Particularly, devices for medical applications such as robot-aided therapy or advanced assistive technologies often struggle to comply with the complex contexts of use, as users with health issues have more specific and personalized needs [7–10].

A solution to tackle and minimize usability limitations is user-centered design (UCD), which aims to involve the technology stakeholders throughout the device development to more successfully address and meet user needs [11–13]. As part of the iterative UCD process, a structured evaluation of the technical solution solving a specific human problem is considered essential [14–17]. The evaluation of WRD is a challenging endeavor with many facets to consider, ranging from technical characteristics to human factors. From a technical perspective, device effectiveness and safety are crucial when developing robots that assist, support, or augment human activities. On the other hand, the users themselves bring physical and psycho-social factors into play, such as varying skills, knowledge, prior experiences or expectations, which need to be investigated and valued as design criteria with equal importance [7, 18]. Unfortunately, there is a lack of standards and guidelines for the evaluation of the complex human-robot interaction of WRD. More specifically, the application of relevant and appropriate usability evaluation measures remains a fundamental challenge in wearable robotics development [19]. Recent studies analyzing WRD evaluation practice have shown a vast landscape of measures being used, with no apparent best-practice identifiable [20–22]. Despite application-specific efforts to address these limitations with novel evaluation frameworks [23–25], there still is a need for more general guidelines on the best-practice for the development and usability evaluation of wearable robots. While the user-perspective remains the key element of WRD usability and technology acceptance [15, 26], it is also crucial to understand how the developers of the technology make the decision of when and how to involve end-users in the design process.

In this work, we report the insights from an online survey sent to developers of WRD from academic, industrial, and clinical backgrounds to analyze current practices in usability evaluation, considering contexts of use, technology maturity and methods used for evaluation. The objectives of the survey were to investigate: (1) whether specific contexts of use for WRD are more advanced in technology maturity than others, (2) whether usability evaluation with active user involvement is current practice in WRD development, as expected from UCD, (3) whether current usability evaluation practices are predominantly device-focused, i.e. limited in capturing the user’s perspective, and (4) whether best-practice for WRD usability evaluation can be inferred from the most frequently reported methods and measures in the field. Our analysis provides a novel understanding of the predominant WRD contexts of use, current technological maturity of the field and current best-practice for usability evaluation. From our data, we propose recommendations that could help harmonize usability evaluation of WRD, and help tackling the acceptance issue of wearable robotics.

## Methods

### Study design

The data for this study was collected with an online survey that was administered using the QuestionPro survey software (QuestionPro Inc., Austin, TX, USA). The survey was designed using predominantly standardized and validated question formats, such as Likert scales and multiple-choice questions, and by using the standardized question formats of QuestionPro. A total of 31 questions covered three topics of interest related to wearable robotics: (a) context of use, (b) technology maturity and user involvement, and (c) usability evaluation practice. The study aims, usage of data and other informed consent information was provided on the survey landing page. Throughout the survey, all technical terms were based on standardized definitions such as provided by the International Organization for Standardization (ISO), as well as supported with additional information provided via a help option. Face validity of the survey questions was established through individual reviews by all authors as well as by a reviewer independent of the research group, who checked for potential leading, confusing, or double-barreled questions during a survey dry run. The total number of questions for each respondent depended on the answers given, as logic branching with follow-up questions was applied. The full survey including all questions is provided in the Additional file 1.

#### Context of use

Building upon definitions of the ISO as terminological ground truth, usability is dependent on the context in which the systems, in our case WRD, are used and investigated. Therefore, we aimed to first understand which contexts of use the collected data represented, such that we could investigate our research questions on development and evaluation practices. The context of use can be specified as “combination of users, goals and tasks, resources, and environment” [6]. The respondents were asked to specify the general usage purpose of their device, alongside of the usage environments, the forms of supervision needed, as well as the target populations.

#### Technology maturity and user involvement

To further understand the technology maturity of their WRD, respondents were asked to specify their Technology Readiness Level (TRL). The nine distinct TRL were introduced as follows, adapted from the Horizon 2020 guideline: TRL 1 = Basic research and principles observed, TRL 2 = Technology concept formulated, TRL 3 = Experimental proof of concept, TRL 4 = Technology tested in lab environment, TRL 5 = Technology tested in intended environment, TRL 6 = Technology validated and demonstrated in intended environment, TRL 7 = Demonstration in operational environment, TRL 8 = System complete and ready for commercialization, TRL 9 = Full commercial application [27]. For the purpose of congregated data analysis, we grouped the TRL into three Technology Readiness Phases (TRP): “Concept” (TRL 1–3), “Prototype” (TRL 4–7), and “Product” (TRL 8–9). Also, respondents were asked to specify the time since project initiation, as a second measure of project maturity. Lastly, the number of users who tested the reported WRD was requested to understand the extent of user involvement, and to validate the TRL estimations. The respondents were thereby asked to distinguish between target users (real end-users) and mock users (neurologically intact controls, team-members, themselves).

#### Usability evaluation practice

A core interest of this study was the investigation of evaluation practices for WRD and, more specifically, of measures used for usability evaluation. From the ISO terminology, we can define usability by the three dimensions effectiveness, efficiency, and satisfaction. Effectiveness reflects “the accuracy and completeness with which users achieve specified goals”, efficiency represents the “resources (time, human effort, costs & materials) used in relation to the results achieved”, and satisfaction is the “extent to which the user’s physical, cognitive and emotional responses that result from the use of a system, product, or service meet the user’s needs and expectation” [6]. In order to understand their evaluation focus, the respondents were asked to distribute their current evaluation efforts as a total of 100% to the three usability dimensions. Although the usability dimensions narrow down the room for terminological interpretation, specific attributes such as comfort, functionality, ease of use, and safety are more frequently used to collect and analyze user feedback or device performance [21]. As one of the aims of this study was to understand how WRD developers define and approach usability evaluation, we provided a list of 34 popular usability attributes (full list in additional materials). Respondents were asked to pick up to five attributes on which they are focusing their usability evaluation. If their preference was not among the listed attributes, new entries were possible. Adapted from the ISO TR 16982–2002, eight specific usability methods were then proposed to the respondents: (1) Performance-related measurement, (2) Questionnaire/Survey, (3) Interview, unstructured oral feedback, (4) Thinking Aloud, (5) Observation of users, (6) Document-based method, (7) Model- or simulation-based approach, (8) (Usability) Expert evaluation [28]. For each previously selected usability attribute, the respondents were asked to state which of these methods were used to assess it. If, at any point, the respondents selected the method types Performance-related measures and/or Questionnaires/Surveys, follow-up questions were generated asking the respondents to specify which exact metric or measure was used. The last few questions of the survey allowed the respondents to reflect on their usability evaluation practice. Respondents were asked to state their level of agreement from 1 (completely disagree) to 5 (completely agree) on usability evaluation usefulness, availability of benchmarks, and preference towards certain types of usability data.

### Sample

The target population of this study were developers of WRD with academic, industrial, and/or clinical backgrounds. Understanding that is the developers, who will decide to what extent a WRD project will follow UCD (i.e., when, and how the end-users will be involved) we dedicated this survey to analyze the developer-perspective. The respondents were instructed to only participate in the survey if they work on, or develop a WRD and to focus on one specific device during survey completion. The survey started after the respondents agreed to the terms and conditions. The survey link was distributed globally using mailing lists, social media (LinkedIn, Twitter), as well as blogs featured on Exoskeleton Report [29] and on Biomed Central [30]. The targeted sample size was 100 fully completed surveys, which consequently provides a larger sample size for questions at the start of the survey (before discontinuation), while guaranteeing a meaningful and diverse sample of WRD projects and their usability practices for questions at the end of the survey. This target sample size was calculated with the dedicated tool provided by QuestionPro and was based on an estimated total target population size of N = 1000, with a 95% confidence interval and a for descriptive analyses accepted margin of error of 9% [31]. Data were collected from June to October 2020.

### Data analysis

The criterion for incomplete responses to be included in the analysis was the minimum completion of survey sections on demographics information, context of use, and user involvement. Responses that did not fulfill the inclusion criteria were removed using the data analysis functions of QuestionPro. The eligible data was exported as EXCEL data reports for post-processing and analysis. All statistical analyses and visualizations were done in MATLAB R2020a (MathWorks, MA, USA) and in RStudio Team 2021 (RStudio PBC, MA, USA). Descriptive statistics such as frequency distributions and cross-tabulations were used to analyze and visualize all ordinal data as from Likert scale questions. For the continuous data generated by the evaluation effort allocation question, Shapiro-Wilk tests were performed for check for univariant normality before applying parametric statistics. After confirmation of normality, paired t-test were used for intra-response comparisons, while for inter-response comparisons a two-sample t-test was used to account for unequal sample sizes in the analyzed subgroups . All statistical tests were performed with a 5% significance level. Locally weighted regression fits (Loess regression) were used for scatter plot smoothing.

## Results

From a total of 286 initiated responses, 158 fulfilled the inclusion criteria and 102 were fully completed (35.6% completion rate). Due to logic-branching and partially missing answers, certain questions had a lower sample number than the total. Therefore, the sample number (n) is provided for each analysis. Out of the 158 respondents, 33.5% were female, and 79.1% were between 25 to 44 years old. The dataset contained WRD projects from all continents except Africa and Antarctica. The background of the respondents ranged from academia (71.5%) to industry (32.3%) and clinical practice (15.2%), while 28 of the 158 respondents selected more than one background. Detailed participant demographics are described in Table 1.

**Table 1:** Respondent demographics (n = 158)

	Frequency	Percentage (%)
Gender		
Male	105	66.5
Female	53	33.5
Age		
18-24	13	8.2
25-34	86	54.4
35-44	39	24.7
45-54	7	4.4
55-64	10	6.3
Above 64	3	1.9
Background^a^		
Academia	113	71.5
Industry	51	32.3
Clinical practice	24	15.2
Location of WRD project		
Europe	88	55.7
North America	37	23.4
Asia	24	15.2
Latin America	7	4.4
Oceania	2	1.3
Africa & Antarctica	0	0.0

^a^More than one background could be selected

### Context of Use

**Fig. 1:**
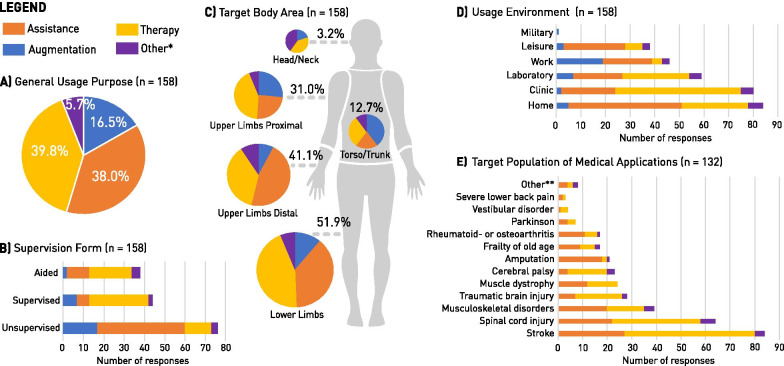
Context of Use of Wearable Robotic Devices: **A** General usage purposes: augmentation, assistance, therapy and other. *Other usage purposes reported were: *all of the above* (n = 3), *brain computer interfaces* (n = 2), *fitness and sports tracking* (n =1), *training and assistance for surgery* (n = 1), *benchmarking* (n = 1) and *user research* (n = 1), **B** Supervision form: We can differentiate between fully supervised, partially-supervised (aided) and unsupervised use of WRD, **C** Target body areas: The relative frequencies of the four general usage purposes are reported for each body area **D** Usage environment: The intended use ranges from rather controlled (laboratory, clinic, home) to more dynamic (leisure, military, work) environments **E** Target population of medical applications: For the medical applications (therapy and assistance) respondents reported the specific target groups. **Other target groups where as: *visually impaired, essential tremor, trauma, rhabdomyolysis, peripheral artery disease, first responders (emergency), physical training and exercise*, and *sports injuries*

The responses covered a large diversity in applications and target user groups. A summary of the context of use information is visualized in Fig. 1. The general usage purposes of the WRD (Fig. 1A) are grouped into four categories: Augmentation (16.5%), Assistance (38.0%) Therapy (39.8%), and Other (5.7%). The grouping of these four general usage purposes was used for most analyses and visualizations. The intended form of supervision visualized in Fig. 1B shows that 76 out of 158 WRD aim for unsupervised, independent use. According to our responses, WRD for therapy applications are intended for use with a certain level of aid (33.3%) or full supervision (46.0%), while the majority of applications for augmentation and assistance are envisioned to be used independently (65.4% and 71.7% respectively).

The most targeted body area by current WRD were the lower limbs (n = 81), out of which 82.7% reported a use for medical applications (Fig. 1C). In the second most reported body area, the upper limbs, we can differentiate between full arm applications (distal + proximal, 18.3%), and specifically distal applications for the hand, wrist and/or lower arm (22.3%). The intended usage environment of the investigated WRD (Fig. 1D) varied greatly depending on the intended usage purpose. While devices for augmentation mostly focus on work applications, assistive WRD aim to also help at home, during leisure activities, and within clinical use. Within the 63 therapy-oriented devices, 80% are designed to be used in clinics with additional usage intentions at home or in research (both 42.8%). Across all reported WRD, the homes of end-users users appeared to be the most targeted usage environment (52.5%) followed by clinics (50.6%) and laboratory (37.3%) applications. Fig. 1E depicts the targeted disorders and disabilities of all WRD for medical applications (n = 132). Stroke survivors built the largest user group across all responses, targeted by 63.6% of all medical WRD. Especially for therapy devices, rehabilitation after stroke is targeted by 85% of all devices. The second largest user group addressed by almost half of all medical applications (48.5%) are people with spinal cord injuries. Out of 132 medical WRD applications, 59.8% are intended for more than one specific population. For example, sensorimotor impairments resulting from stroke or spinal cord injury are often targeted with the same WRD.

### Technology maturity and user evaluation

**Fig. 2:**
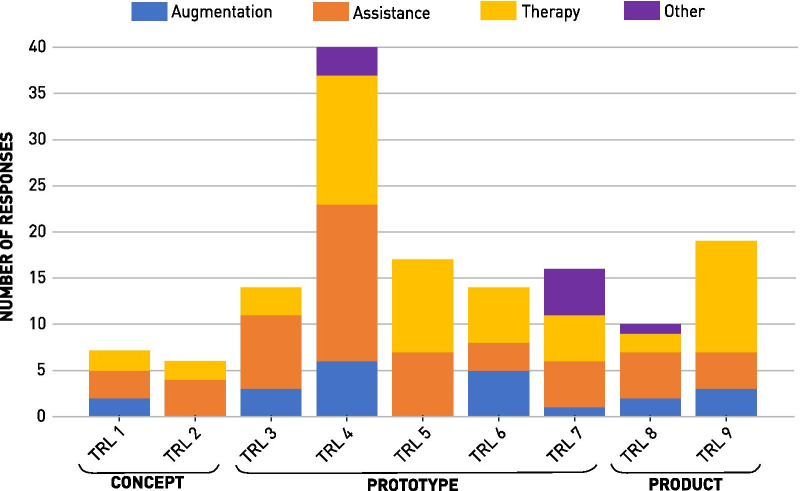
Technology Maturity of Current Wearable Robotic Devices: The Technology Readiness Levels (TRL) of the four general usage purposes, visualized as stacked histogram (n = 143). The TRL were grouped in three Technology Readiness Phases (TRP): “Concept” (TRL 1–3), “Prototype” (TRL 4–7) and “Product” (TRL 8–9)

The TRL distribution of 143 WRD is visualized in Fig. 2. The four usage purposes showed a similar distribution across the TRL spectrum. The largest total number of WRD (n = 40, 28.0%) classified as TRL 4. Ordered by the mean TRL as usage purpose maturity indicator, the most market ready applications are Other (6.11 1.62) and Therapy (5.64 2.29) followed by augmentation (5.23 2.43) and Assistance (4.77 2.18). Overall, 60.8% of the reported WRD were classified as prototypes, 20.3% as products, and 18.9% as concepts.

**Fig. 3:**
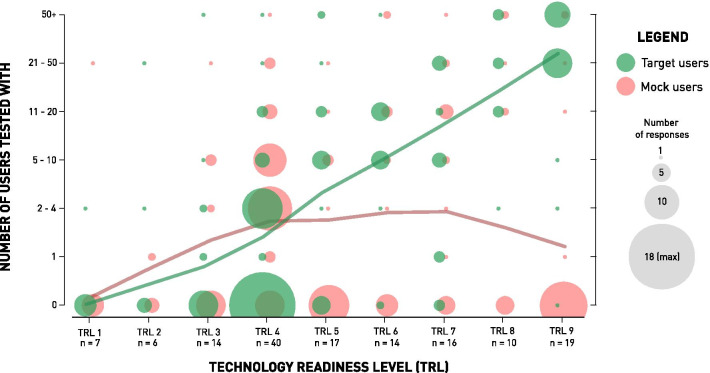
Progression of User Involvement with Technology Maturation. The number of users (target and mock) per Technology Readiness Level (TRL) are shown (n = 143). The size of the circles represents the number of responses within each TRL. For all respondents that only indicated testing with the one specific user group (target or mock), 0 users are shown in the opposite group. The Loess regression lines were fitted to smooth the scatter plot visualization

In terms of project duration, 41.9% of WRD projects were initiated within the last 2 years preceding survey completion, while 23.2% have been ongoing for more than 5 years. Of all 158 WRD projects recorded, 108 (68.4%) tested their device with at least one target user so far. From those 108, 48.1% additionally tested with mock users. No tests with the target population, but only with mock users was performed in 17.1% of the 158 WRD. Overall, 125 of 158 WRD (85.4%) reported some sort of user testing. Fig. 3 shows the number of tested users, according to the TRL reported (n = 143).

### Usability evaluation practice

**Fig. 4:**
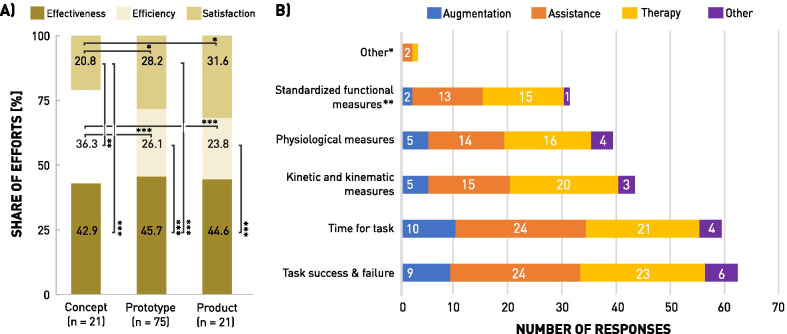
Analysis of Usability Evaluation Practice: **A** Evaluation efforts allocation per Technology Readiness Phase (TRP, n = 117): The allocated total of 100 evaluation-effort-points among the usability dimensions effectiveness, efficiency, and satisfaction, per TRP are shown. Paired comparison between the three dimension were analyzed within each TRP, while unpaired, two-sample comparisons between the TRP were calculated. Levels of significance indicated as: * = p < 0.05, ** = p < 0.01, *** = p < 0.001. **B** List of reported performance-related measurements (PRM, n = 88): *Other PRM described as *spatiotemporal metric analysis*, *eye-tracking recording*, *number of steps*. **Standardized functional measures (SFM) specified: *upper limb SFM* Box and Block Test (BBT, n = 6), Jebsen-Taylor Hand Function Test (JTHFT, n = 5), Action Research Arm Test (ARAT, n = 4), Chedoke Arm and Hand Activity Inventory (CAHAI, n = 2), Southampton Hand Assessment Procedure (SHAP), Assisting Hand Assessment (AHA), Smart Pegboard, Frenchay Arm Test (FAT), *lower limb SFM* 10 Meter Walk Test (10MWT, n = 2), 2 Minute Walk Test (2MWT, n = 2), 6 min Walk Test (6MWT, n = 2), Timed-up-and-Go (TUG), *general SFM* Human-Robot Fluency Metrics, Assessment of Capacity for Myoelectric Control (ACMC), Thermography, Failure Mode and Effects Analysis (FMEA), ISO regulation, fit and tolerance assemblies

The results of the usability evaluation efforts allocation (n = 117) are shown in Fig. 4A. The paired comparisons between the usability dimensions within each Technology Readiness Phase (TRP) showed significant differences in terms of efforts allocations: in the conceptual phase, significantly fewer evaluation effort was dedicated to satisfaction compared to effectiveness (p < 0.001) and efficiency (p < 0.001). When evaluating prototypes, effectiveness remained the most dominant usability dimension over satisfaction (p < 0.001) and efficiency (p < 0.001). In the product phase, only the difference of evaluation efforts split between effectiveness and efficiency showed a significant difference (p < 0.001). In the unpaired, two-sample comparison of each usability dimension evaluation, we can observe that satisfaction gained focus in the prototyping (p < 0.05) and product (p < 0.05) phases compared to the conceptual phase. In contrast, the relative efforts to evaluate efficiency appeared reduce as technology matures (concept to prototype, p < 0.001; concept to product, p < 0.001). The share of effectiveness in the evaluation efforts of WRD remained constantly high between 42.9–45.7%. Details of individual p-values, as well as additional analyses of evaluation effort allocations between other data groups such as form of supervision and general usage purpose are listed in the Additional file 3.

A frequency analysis of the top 15 selected usability attributes (ordered from most to least selected) and their respective evaluation methods is summarized in Table 2. The most frequently evaluated usability attributes were functionality (37.6%), ease of use (36.8%), performance (32.0 %), safety (32.0 %), and comfort (29.6%). The least selected attributes were learnability (4.0%), mental demand (3.2%), and understandability (2.4%). The most reported usability evaluation method across all attributes are performance-related measurements (PRM), followed by questionnaires and observation of users, while the least used appear to be Thinking Aloud and document-based methods. PRM were reported to be used to evaluate almost all of the listed usability attributes (94.1%) except for complexity and mental demand. Attributes of a more subjective nature, such as comfort, ergonomics, wearability, adaptability, and intuitiveness appear to be primarily evaluated with qualitative measures (unstructured interviews, observations) and questionnaires/surveys. The full table with all 34 attributes is provided in Additional file 2.

**Table 2:**
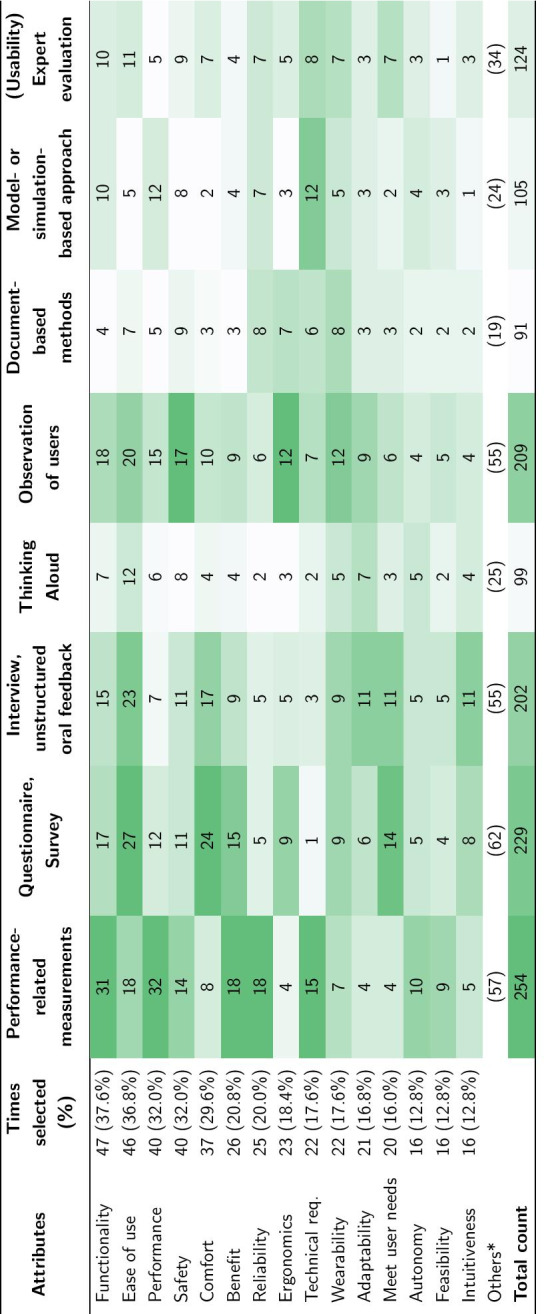
Usability attributes and evaluation methods (n = 125)

*Full list of attributes in Additional file 2

Figure 4B shows the selection frequencies of specific PRM, ordered from most to least used. Task success & failure (70.5%) and time for task (67.0%) were reported as the most popular PRM across all general usage purposes. From the provided selection of specific PRM, standardized functional measures, such as the 10 Meter Walk Test or the Box and Block Test were reported to be used the least (35.2%). The highest relative usage of standardized tools was observed for medical applications. Kinetic and kinematic measures such as interaction forces, or spatiotemporal parameters appear to be most valued in the evaluation of WRD for therapy.

**Fig. 5:**
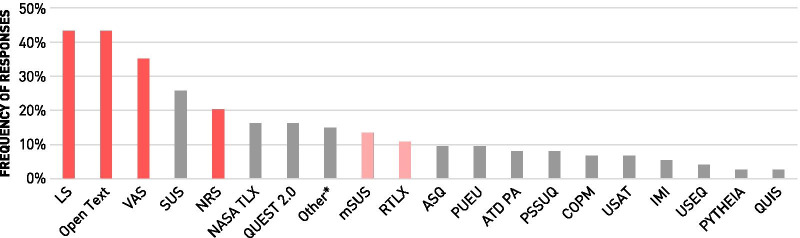
Questionnaires and Scales Used in Wearable Robotics Usability Evaluation: The 20 most frequently selected questionnaires and scales are displayed (n = 74). Custom-made forms are marked in dark red, modified questionnaires are marked in light red. Abbreviations: LS = Likert Scale, VAS = Visual Analogue Scale, SUS = System Usability Scale, NRS = Numeric Rating Scale, NASA TLX = NASA Task Load Index, QUEST 2.0 = Quebec User Evaluation of Satisfaction with Assistive Technology, mSUS = modified System Usability Scale, RTLX = Raw Task Load Index, ASQ = After Scenario Questionnaire, PUEU = Perceived Usefulness, Perceived Ease of Use, ATD PA = Assistive Technology Device Predisposition Assessment, PSSUQ = Post-Study Usability Questionnaire, COPM = Canadian Occupational Performance Measure, USAT = Usability Scale for Assistive Technology, IMI = Intrinsic Motivation Inventory, USEQ = Usefulness, Satisfaction, Ease of Use Questionnaire, PYTHEIA = Psychometric Scale to Assess the Satisfaction of Users with Assistive Technology, QUIS = The Questionnaire for User interaction Satisfaction. *Other = Borg Scale of Perceived Exertion, Michigan Hand Outcomes Questionnaire, Prosthesis Evaluation Questionnaire, Psychosocial Impact of Assistive Device, Questionnaire to Explore Human Factors and their Technical potential, QuickDASH, SF-36, Telehealthcare Satisfaction Questionnaire - Wearable Technology, Trinity Amputation and Prosthesis Experience Scale, Usability Metric for User Experience, and Embodiment Questionnaire

The results from 74 respondents who specified which questionnaires and scales they use are summarized in Fig. 5. Custom-made questionnaires made with Likert Scales (LS), Visual Analogue Scales (VAS), Numeric Rating Scales (NRS), or open text questions were most frequently reported. Overall, 73.0% of all respondents indicated the use of at least one custom-made questionnaire. Still, most evaluations protocols appear to be a mix of standardized and custom-made measures, as only 25.7% of the respondents indicated the sole use of custom tools. From 74 responses, 22 (29.7%) only chose one specific measure, while 38 (51.3%) selected three or more from the provided list or added additional ones specified in “Other”. The System Usability Scale (SUS, 25.7%), NASA Task Load Index (TLX, 16.2%) and Quebec User Evaluation of Satisfaction with Assistive Technology 2.0 (QUEST 2.0, 16.2%) represent the most frequently selected standardized and validated questionnaires. Modifications of standardized scales are also frequently used, as the modified SUS and the raw, unweighted TLX were both selected by more than 10% of the respondents.

**Fig. 6:**
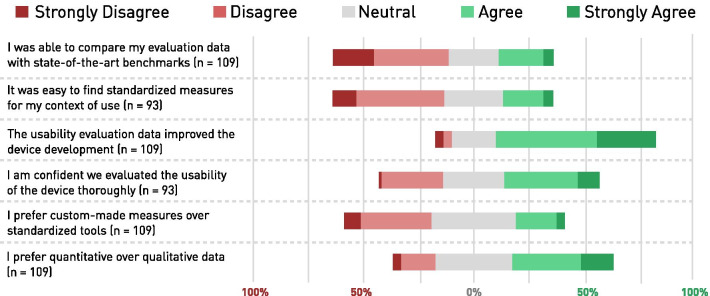
Reflection of Usability Evaluation Practise: The level of agreement for each statement is shown. Disagreement is red, agreement is green. The more the entire bar (100%) is shifted to the left or right, the clearer a trend of agree- or disagreement is observable among the responses

Lastly, the insights from the reflections on personal usability evaluation practices are summarized in Fig. 6. More than half of all WRD developers showed a certain level of disagreement with the two statements “I was able to compare my evaluation data with state-of-the-art benchmarks” (52.3% disagreement, n = 109) and “It was easy to find standardized measures for my context of use” (50.5% disagreement, n = 93). From the 109 respondents that completed this last part of the survey, 72.5% agreed that usability evaluation improved their WRD. When asked if they would prefer custom-made measures over standardized tools, most participants selected the neutral option. The respondents showed the second lowest level of disagreement with the statement “I prefer quantitative over qualitative data” (18.9% disagreement, n = 109).

## Discussion

The aim of this work was to investigate current landscape in WRD applications, the extent of their user-centered design practice, as well as their usability evaluation practices. We collected information on contexts of use, technology maturities, and usability evaluation strategies from a heterogeneous population of 158 WRD developers to provide an analysis of trends and best-practices for the development of wearable robotics.

### Contexts of use and maturity of wearable robots

The proposed breakdown of general usage purposes, target users, usage environments, and supervision forms provides a realistic glimpse into the current application landscape of wearable robotics. From the respondents’ data, we can conclude that applications for robot-aided therapy and daily assistance of people with functional disabilities currently build the largest use cases of WRD. In combination with the TRL information, we understand that devices for daily-life assistance are in a comparably younger maturity state compared to the other usage purposes. Robot-aided therapy appears to be more mature overall, with a larger number of devices close to—or already available on the market. In our collected sample, devices for augmentation appear to be a minority of WRD use cases, with only 16.5% of the investigated devices aiming for, e.g., prevention of work-related musculoskeletal disorders in industrial applications. Most reported augmentation solutions target to support the torso (e.g., support lifting tasks) or proximal upper limbs (e.g., overhead work). Combining all insights from the context of use data, we can identify the three currently most popular contexts of use in the WRD field, ranked by their current technology maturity and success in translation to daily use: (1) Robot-aided therapy applications for the upper and lower extremities in a supervised (clinical or research) environment, (2) Augmentation for labor-intensive tasks in industrial, unsupervised workplaces, and (3) Unsupervised, independent use of wearable robotic assistive technology in the home environment.

Even though the WRD field initially emerged from industrial, i.e., augmentation applications for the upper limbs [32], the predominant number of lower limb WRD is no surprise. A majority of WRD developments and research of the past two decades focused on the challenge of restoring or assisting human gait [1, 3]. Pioneering devices such as the robotic-driven gait orthosis Lokomat [33] have triggered a shift from initially more augmentation-oriented towards medical use cases, demanding a more user-centered focus on human factors [15, 32]. However, WRD for upper limb, and torso augmentation have regained focus in the last years, as more lightweight and simple solutions using passive, or semi-active actuation principles have found their niche in industrial scenarios [5]. Also, robotic hand orthoses for both, assistance and therapy, have gained momentum in the last decade. Interestingly, a recent review on the technological maturity of such hand orthoses provided very similar insights in terms of TRL which indicates that our data might realistically represent the current WRD maturity state [34]. In both analyses, TRL 4 (= technology tested in lab environment) appears to be a bottleneck for numerous WRD developments, indicating the first implementation barrier between basic research and application, also known as “valley of death”. Developments beyond TRL 4 are likely to exceed the basic research questions of academia, and the proof-of-concept required for technical publication. An unfortunate consequence is that the majority of promising WRD projects never make their way out of laboratory research, which also requires additional substantial resources. By supporting more application-oriented, translational research, academia could push WRD maturity towards TRL 6 or 7, and overcome the translational gap to materialize the potential of wearable robotics [35].

Robotic assistive technology was reported with an overall lower technology maturity than all other usage purposes, which in parts contradicts the rich history of upper and lower limb prosthetics as assistive mobility devices. This observation might be explained by terminological inconsistencies, as the term WRD is often used as a synonym for exoskeletons [32]. Exoskeletons are robotic orthoses, which so far showed rather limited technology acceptance in unsupervised, assistive applications [36, 37]. Defining WRD and exoskeletons as synonyms would therefore wrongfully exclude prosthetics, which in fact make up 43% of all emerging mobility assistive technologies, while exoskeletons only occupy 19% of that specific market [38]. Prosthetics—and their advanced technology readiness—might thus be under-sampled in our dataset (22 out of 158 responses).

Another point worth highlighting is the form of supervision, which has been only minimally discussed in existing WRD reviews [1, 4, 5]. Here, our data indicates that, e.g., WRD for therapy might be a mature WRD technology due to the fact that their (intended) application is a controlled environment under the supervision or aid of trained personnel. Hence, WRD for therapy are mainly used and acquired by institutions such as rehabilitation facilities, while WRD for assistance and augmentation aim towards independent use by individuals at home, work, or for leisure activities. This implies that WRD for assistance and augmentation have to work reliably in uncontrolled and more dynamic environments. We can therefore argue that one factor limiting the usability, acceptance, and translation of WRD for augmentation and assistance appears to be their goal of unsupervised, independent use. Moreover, the complexity of WRDs appears to be among a strong limiting factors of technology adoption, even for a supervised use in therapeutic applications [39]. Developing a robotic device with high functionality while keeping the design simple enough to be set up, used and maintained remains a central challenge in WRD. A strong development focus on UCD and usability evaluation could help overcoming this challenge.

### User involvement and evaluation focus

The first step towards UCD is active user involvement. Although users should be involved in all development phases, this survey focused on the evaluation phase. More specifically we analyzed the number of users our respondents have tested, i.e. evaluated their WRD solution with, and which methods they used in this process. Our results show that the number of target users involved increases steadily with technology maturation. Testing with mock users reaches a plateau in the prototype stage, but over-weights target user involvement until TRL 4, also because almost half of all projects claiming TRL 4 maturity did not test with a single target user yet. This practice may likely come from the circumstances that target users are only involved once a WRD concept has proven effective, and that certain regulatory, or safety measures limit the access to target users. Also, limited resources—as discussed above—might be an additional factor limiting target user involvement in early development stages. However, also a substantial amount of WRD projects in the later prototyping stages (TRL 5, 6, 7) reported to have involved less than five target users, which might explain usability issues encountered when eventually aiming for commercialization. This was somewhat confirmed by the evaluation efforts allocations, as we learned that effectiveness remains the primarily investigated usability dimension across the entire technology maturity continuum. Satisfaction, which is by nature a more target user-focused value, only gains focus once the WRD comes closer to the product stage, and efficiency appears to even loose focus as technology matures. These insights suggest that a large number of WRD projects involve target users at a later stage only, while first focusing on the effectiveness of their solution.

The practice of device-oriented development and evaluation has been previously highlighted by works of Contreras-Vidal et al. and Pinto-Fernandez et al. [22, 40] who both summarized that in lower limb exoskeletons studies, evaluation outcomes on comfort, ergonomics, satisfaction, and/or mental demand are drastically scarce compared to performance-related outcomes. In a similar survey exploring user involvement and device evaluation strategies in lower limb specific projects, Ármannsdóttir et al. [21] also identified functionality as the most frequently assessed aspect of usability. An evaluation focus on WRD effectiveness seems somewhat rational—especially in early development stages—as developers first want to make sure the device does what it is supposed to do, and this in a safe, reliable, and accurate way. Another reason for the higher prevalence of device-oriented developments and their reporting in literature may come from the observations that qualitative metrics are less well-accepted as outcomes of WRD usability studies. The potential reliability, reproducibility and biasing limitations when reporting subjective outcomes could be factors contributing to their limited selection, or under-reporting as scientific methods. Indeed, with our survey we could identify an apparently unpublished, but nonetheless frequent use of user-oriented evaluation. Attributes such as ease of use, safety and comfort appear to be often evaluated, indicating a frequent practice of qualitative user evaluation. Such user-oriented evaluation from early on is expected to enhance stakeholder involvement and may help meeting user needs right from the start [37, 41, 42]. We can conclude that while effectiveness may remain a core focus of development and evaluation of WRD, at least 50% of all efforts should be directed towards optimizing efficiency and satisfaction from early on. The importance of functional performance and device effectiveness is indubitable, but at the end of the day, it is the overall user experience that decides whether a device will be used in daily life or not.

### Usability measures and methods

In our detailed analysis of specific measures used in WRD usability evaluation, the surprisingly frequent use of user-oriented methods was further revealed. All eight types of usability evaluation methods as listed by ISO TR 16982–2002 were reported to be used [28]. Most projects report a combination between objective and subjective evaluation, with methods such as questionnaires, interviews, and observations complementing PRM as most popular methods. Simple and fast quantitative usability data, mostly collected with PRM such as task success & failure, appear to be preferred in the WRD field, which is in line with the effectiveness-driven focus discussed above. Standardized functional measures such as clinical-grade function tests were the least reported PRM, mainly because context-specific, standardized measures are hard to find and less preferred than customized ones. A similar trend was observed for the use of questionnaires and scales, were we found that 73% of all WRD projects use custom-made measures, often in combination with standardized ones such as the SUS.

These findings can be partially validated with recent works investigating robotic assistive technology evaluation, which comparably showed that custom-made usability measures are used in 60–70% of all reported studies [20, 21]. Also, we could extend the findings from Koumpouros [20], who previously listed the SUS, QUEST 2.0, and NASA TLX as the most frequently used standardized questionnaires to subjectively measure WRD usability. The general preference for easy-to-administer, objective PRM such as time for task or task success & failure was also observed by Pinto-Fernandez et al. [22]. Their review focusing on lower limb exoskeleton evaluation also highlighted the popularity of kinetic and kinematic measures, which was further confirmed by a recent review on soft wearable robotics by Xiloyannis et al. [3]. Furthermore, physiological measures such as muscle activation, heart rate, or metabolic consumption are frequently reported measures of WRD usability, especially among the uprising soft robotic technologies [3].

Although certain popular measures are validated and generalizable, an overall preference towards custom tools is a clear limiting factor in the current usability evaluation practice of WRD. This appears to be a chicken-and-egg problem: WRD developers can’t find validated and standardized tools for their specific context of use and thus start creating their own, customized measures. At the same time, the usage of such non-standardized and non-validated metrics inhibits the emergence of evaluation standards and benchmarks. Another factor that fuels this problem is the generally scarce availability of WRD usability studies. As highlighted in a 15 years reflection on the papers published in the Journal of Neuroengineering and Rehabilitation, only 2.4% of the works published included the term “usability” [2]. Of those studies, only very few report qualitative data, which is further limiting the body of literature on this topic. What the WRD field therefore might need, are not only guidelines and benchmarks for usability evaluation practice, but an agreement among peers to also value qualitative research as scientific practice worth publishing.

### Limitations and implications

The insights and conclusions generated from this survey data should be taken with care, as a sample size of 158 responses may not fully capture the diversity of the WRD field. Also, the understanding and judgement of specific information such as TRL, or number of users tested with, might differ, depending on the respondent’s role in the WRD development (e.g., clinical collaborator). In general, a certain response bias, i.e., untruthful, or inaccurate answers is to be expected in any kind of online survey. In addition, a potential selection bias might limit the generalization of our data, despite our efforts to distribute the survey not only via the authors’ networks, but also interdisciplinary, global platforms. It can thus be argued that specific context of use, or usability evaluation practices might be somewhat underrepresented in this study. Lastly, it is important to state that additional, not specifically investigated development factors such as regulatory affairs, or ethical and cultural differences might influence the WRD design and evaluation in our global sample. Despite these limitations, we believe that our global, heterogeneous study sample allowed a novel understanding and comprehensive overview of today’s WRD applications, their development stages and current usability evaluation practices. We could further elucidate the need for evaluation benchmarks and development guidelines, as it has been detailed and called for in recent works [19, 25]. Our analysis may even support the benchmarking endeavors of initiatives such as the EUROBENCH project [43], the Exo Technology Center of Excellence from ASTM International [44] or the CYBATHLON [45].

### Evaluation recommendations

From the insights generated through this survey, we can propose recommendations for technology developers on when and how to collect user feedback in order to support the UCD of wearable robots:

1. We recommend to consciously distribute evaluation efforts between the three usability dimensions effectiveness, efficiency, and satisfaction. While this distribution will likely adapt as technology matures (see Fig. 4a), it is important to consider efficiency and satisfaction from the very start of a WRD project. This implies that developers should be inclined to include target users as soon as possible, also the early stages of WRD development (TRL 1–3, see Fig. 3). A simple walk-through, interviews, or focus groups with target users can shape your concept or low-fidelity prototype in the right direction from the start.
2. Our survey showed that, in contrast to what is mainly reported in literature, a usability evaluation protocol should include substantial amounts of qualitative measures (see Table 2 and Fig. 6). While quantitative data allows for comparison to previous design iterations and the state of the art, qualitative data helps identifying usability issues that may not be obvious to WRD developers when only collecting and comparing numbers. Also, qualitative evaluation by nature calls for more personal interaction with the target users and their satisfaction with the device, thus further promoting the endeavors of recommendation 1.
3. The often necessary usage of custom-made or modified measures to investigate specific research questions should optimally be complemented with standardized tools to allow a fair outside comparison limiting evaluation bias. We could confirm that the current ratio of customized-to-standardized usability measures is roughly 2:1 (see Figs. 4b and 5). We recommend reversing this ratio, by dedicating two-thirds of a usability evaluation protocol to reproducible, standardized measures. This may likely increase data validity and generalizability and could further help the wearable robotics field to establish evaluation benchmarks.

These recommendations, together with the detailed breakdown of methods and measures used, may help WRD developers to define their usability evaluation protocols and to understand which measurement tools could fit their own context of use. This survey data will further be used for the development and data-driven training of a usability evaluation toolbox for WRD [46], which will allow the users to simply enter their context of use to find a list of validated and relevant measures together with our evaluation guidelines.

## Conclusions

This study provided insights into the current evaluation practices and specified the contexts of use of wearable robots for human augmentation, assistance and/or therapy. Evaluation protocols across the various applications are similar, but the lack of applicable guidelines restricts the validation and benchmarking of usability standards in the field of wearable robotics. The individual adaption of evaluation protocols to specific contexts of use remains a fundamental barrier and challenge to get more reproducible evaluation data to push forward user-centered designs of wearable robotic devices. The insights generated by this survey might serve as a data-driven basis for evaluation protocol recommendations and help researchers in defining or comparing their evaluation protocols and data. We believe that more structured and comparable usability evaluations can tackle the technology acceptance limitations of WRD and can help making first steps towards WRD evaluation benchmarking to eventually fill the translational gap of wearable robotics.

## Supplementary information

**Additional file 1.** Print of of full survey.

**Additional file 2.** Extension of Table 2 with all usability attributes.

**Additional file 3.** Figure S1 and Tables S1 and S2.

## Data Availability

Data and materials can be made available upon reasonable request to the authors.

## Abbreviations

WRD: Wearable Robotic Devices
UCD: User-centered Design
TRL: Technology Readiness Level
TRP: Technology Readiness Phase
PRM: Performance-related Measures
SFM: Standardized Functional Measure
LS: Likert Scale
VAS: Visual Analogue Scale
SUS: System Usability Scale
NRS: Numeric Rating Scale
NASA TLX: NASA Task Load Index
QUEST 2.0: Quebec User Evaluation of Satisfaction with Assistive Technology
mSUS: modified System Usability Scale
RTLX: Raw Task Load Index
ASQ: After Scenario Questionnaire
PUEU: Perceived Usefulness, Perceived Ease of Use
ATD PA: Assistive Technology Device Predisposition Assessment
PSSUQ: Post-Study Usability Questionnaire
COPM: Canadian Occupational Performance Measure
USAT: Usability Scale for Assistive Technology
IMI: Intrinsic Motivation Inventory
USEQ: Usefulness, Satisfaction, Ease of Use Questionnaire
PYTHEIA: Psychometric Scale to Assess the Satisfaction of Users with Assistive Technology
QUIS: The Questionnaire for User interaction Satisfaction
MHQ: Michigan Hand Outcomes Questionnaire
PEQ: Prosthesis Evaluation Questionnaire
PIADS: Psychosocial Impact of Assistive Device
QEFTH: Questionnaire to Explore Human Factors and their Technical potential
TSQ-WT: Telehealthcare Satisfaction Questionnaire-Wearable Technology
PIADS: The Psychosocial Impact of Assistive Devices Scale
TAPES: Trinity Amputation and Prosthesis Experience Scale
UMUX: Usability Metric for User Experience
