# Supplementary material for: An analysis of usability evaluation practices and contexts of use in wearable robotics
Source: J Neuroeng Rehabil. 2021 Dec 9;18:170. doi: 10.1186/s12984-021-00963-8 (PMC8656061; doi:10.1186/s12984-021-00963-8)
Supplement: Supplementary file 1 — Additional file 1. Print of of full survey. [file 12984_2021_963_MOESM1_ESM.pdf]

SUPPLEMENTARY MATERIALS

An Analysis of Usability Evaluation Practices and Contexts of Use in Wearable Robotics

Jan T Meyer [1*], Roger Gassert[1,2] and Olivier Lambercy[1,2]

[1] Rehabilitation Engineering Laboratory, Department of Health Sciences and Technology, ETH Zurich, Switzerland

[2] Future Health Technologies, Singapore-ETH Centre, Campus for Research Excellence And Technological Enterprise (CREATE), Singapore

[*]Correspondence:
relab.publications@hest.ethz.ch

Full list of author information is available in the main text file

Additional file 1 (pages 2 - 23) - Full survey print from QuestionPro interface

Additional file 2 (page 24 - 25) - Extension of Table 2 with all usability attributes

Additional file 3 (page 26 - 27) - Figure S1 and Table S1, S2

Additional file 1

Full survey print from QuestionPro interface.

ETH zürich DHEST Department of Health Sciences and Technology RELAB Rehabilitation Engineering Lab

Usability Evaluation Survey for Wearable Robots

Thank you for participating in our online survey. The data collected will be used to **investigate the current practices and limitations of usability evaluation** as part of the human-centered design process for wearable robotics. The insights will help us to find new guidelines and methods to support these endeavors.

If you work on multiple wearable robots, we'd like you to please focus on the current state and evaluation of **one project/device only**. This helps us to relate the **context of use** to the survey results. You can, if you want, complete the survey multiple times to cover other projects/devices.

To fully complete the survey, **approximately 10 - 15 minutes are required**. If you need additional information to understand a question, please click on the question mark (?) when provided.

This work is supported by the Vontobel Foundation and the Swiss National Science Foundation through the National Centre of Competence in Research on Robotics. The data will be stored anonymously on the secured servers of QuestionPro (temporary) and ETH Zurich (permanently) with access limited to the survey coordinators. The anonymized data may be shared and made public. By clicking **"Next"** below, you agree to these terms and conditions.

For further questions, contact us directly via mail at: jan.meyer@hest.ethz.ch

Thank you in advance!

Please check the box to proceed to the survey

☐ I'm not a robot

reCAPTCHA
Privacy - Terms

* What is your **age**?

- ◯ Under 18
- ◯ 18-24
- ◯ 25-34
- ◯ 35-44
- ◯ 45-54
- ◯ 55-64
- ◯ Above 64

* What is your **gender**?

- ◯ Male
- ◯ Female
- ◯ Prefer not to disclose
- ◯ Prefer to self-describe

* What is your **current career path**?

You can also choose multiple options

- ☐ Academia
- ☐ Industry
- ☐ Clinical practice

* What is your **current academic position**?

- ◯ Student (graduate: MSc or undergraduate: BSc)
- ◯ PhD candidate
- ◯ Post-doctoral fellow or senior researcher
- ◯ Professor (assistant, associate or full)
- ◯ Other

Where is your work on wearable robots (mainly) located?

Africa

Asia

Europe

Oceania

North America

Central America or Caribbean

South America

* What is the **primary purpose** of your wearable robotic device?

- ◯ Augmentation & support
  E.g. industrial or military application for neurologically intact users
- ◯ Assistance in daily living
  E.g. orthotics and prosthetics for neurologically impaired users
- ◯ Robot-aided/assisted therapy
  E.g. minimally-supervised therapy for neurologically impaired users
- ◯ Other

---

* How would you best **describe your assistive device**?

- ◯ Active/powered orthosis
- ◯ Passive/body-powered orthosis
- ◯ Active/powered prosthesis
- ◯ Passive/body-powered prosthesis
- ◯ Other

---

* Is your device development somehow related to the **CYBATHLON**?

Click here to learn more about the event: (?)

- ☐ Yes, we aim to participate in 2020
- ☐ Yes, we participated in 2016
- ☐ No, our development is unrelated to the event
- ☐ Other

---

* How would you best **describe your robot-aided/assisted therapy device**?

- ◯ Fully wearable, mobile device
  (all components of the device are wearable and the device is fully mobile)
- ◯ Partially wearable, stationary device
  (certain components of the device are wearable, but the main device remains stationary)
- ◯ Other

* What **body part(s)** does your device augment/support/assist/give therapy to?

You can choose multiple options to e.g. enter whole-arm applications

Upper limb(s) - proximal

Upper limb(s) - distal

Lower limb(s)

Torso/trunk

Head and/or neck

* In what **age group(s)** is/are your primary end-user(s)?

You can choose multiple options

- [ ] Children & adolescent
  0 - 17 years
- [ ] Young adults
  18 - 34 years
- [ ] Middle age adults
  35 - 64 years
- [ ] Elderly adults
  65+ years

* Which **target group(s)** do you (want to) assist or give therapy to?

People with...

- [ ] Amputation
- [ ] Cerebral palsy
- [ ] Frailty of old age
- [ ] Multiple sclerosis
- [ ] Muscle dystrophy
- [ ] Musculoskeletal disorders
- [ ] Osteoarthritis
- [ ] Parkinson
- [ ] Rheumatoid arthritis
- [ ] Severe lower back pain
- [ ] Spinal cord injury
- [ ] Stroke
- [ ] Traumatic brain injury
- [ ] Vestibular disorder
- [ ] Other

---

* With **what kind of users** did you test your device?

You can choose multiple options

- [ ] Real end-users
- [ ] Mock-users
  E.g. team-member, volunteer, yourself
- [ ] No user tests (yet)

* With **how many real end-users** have you tested your device?

- ◯ 1 user
- ◯ 2 - 4 users
- ◯ 5 - 10 users
- ◯ 11 - 20 users
- ◯ 21 - 50 users
- ◯ more than 50 users

---

* With **how many mock-users** have you tested your device?

- ◯ 1 user
- ◯ 2 - 4 users
- ◯ 5 - 10 users
- ◯ 11 - 20 users
- ◯ 21 - 50 users
- ◯ more than 50 users

---

* What is your device's (mainly) **intended usage environment**?

You can choose multiple options

- ☐ Laboratory/clinical research
- ☐ Clinical practise
- ☐ Home
- ☐ Leisure
- ☐ Work
- ☐ Other

* What **form of usage** do you aim for?

- ◯ Supervised/dependent use with trained personnel
- ◯ Aided/assisted use with intructed helper (caregiver, family, colleague etc.)
- ◯ Unsupervised/independent use

* **How long ago** was your device/project initiated?

- ◯ less than 6 months ago
- ◯ 6 - 12 months ago
- ◯ 1 - 2 years ago
- ◯ 2 - 3 years ago
- ◯ 3 - 4 years ago
- ◯ 4 - 5 years ago
- ◯ More than 5 years ago

* What is the **current Technology Readiness Level (TRL)*** of your device:

If you are unsure or in between two levels, please select the lower level.

* Figure adapted from Horizon, 2020

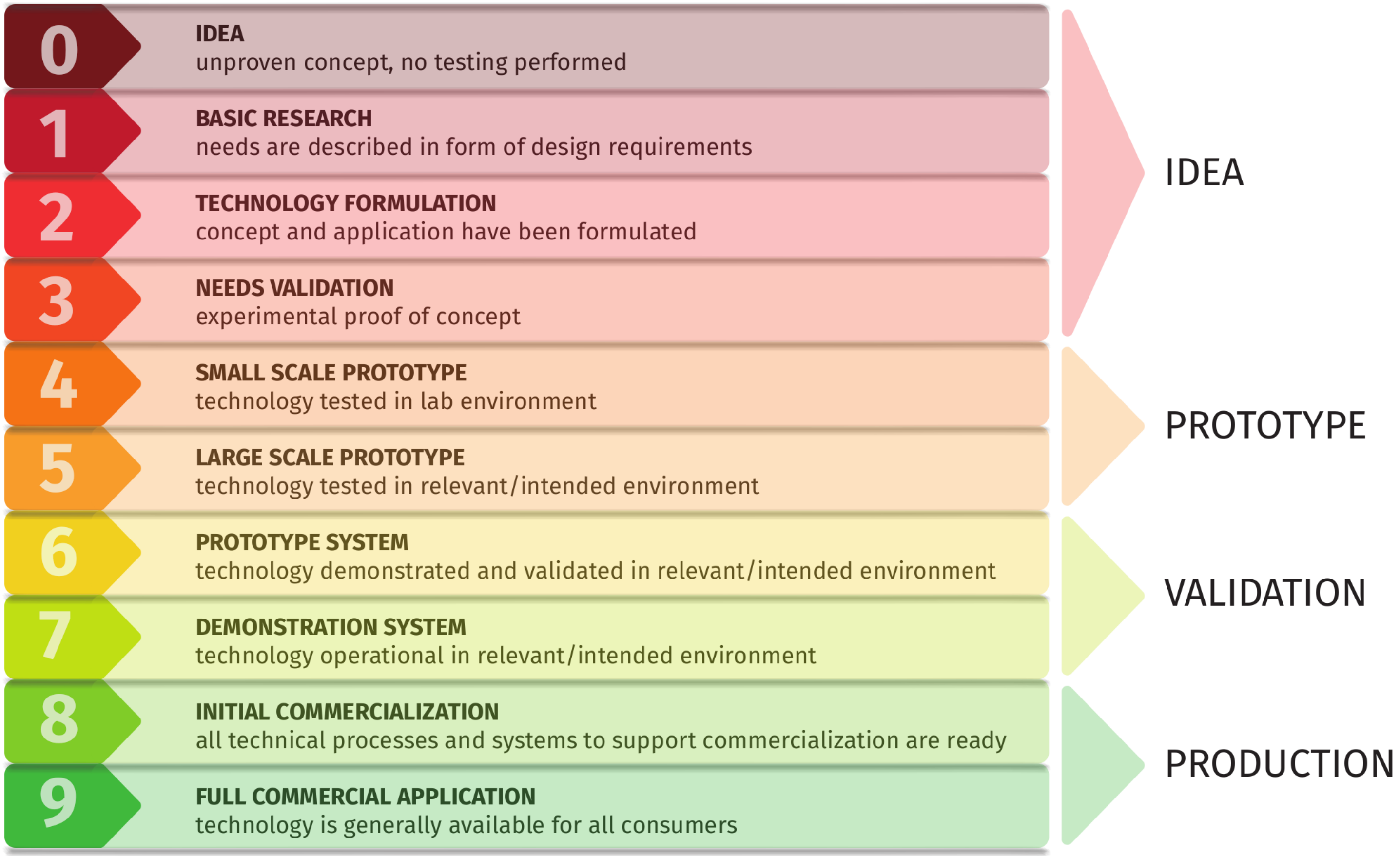

- ◯ TRL 0
- ◯ TRL 1
- ◯ TRL 2
- ◯ TRL 3
- ◯ TRL 4
- ◯ TRL 5
- ◯ TRL 6
- ◯ TRL 7
- ◯ TRL 8
- ◯ TRL 9

To answer the following questions, please consider the following definition of usability according to ISO 9241-11:2018 (EN):

usability *= "the extent to which a system, product or service can be used by specified users to achieve specified goals with* ***effectiveness****,* ***efficiency,*** *and* ***satisfaction*** *in a specified context of use"*

- effectiveness = accuracy and completeness with which users achieve specified goals
- efficiency = resources (time, human effort, costs & materials) used in relation to the results achieved
- satisfaction = extent to which the user's physical, cognitive and emotional responses that result from the use of a system, product or service meet the user's needs and expectations

* **How important** do you consider the listed usability dimensions to be in **your current Technology Readiness Level** (TRL)?

0 = not important at all, 10 = extremely important

?

Effectiveness

Efficiency

Satisfaction

Please allocate a total of 100 points on **how you currently spend your efforts** in evaluating the listed usability dimensions:

You can also directly type in a number in the boxes

?

Effectiveness

Efficiency

Satisfaction

0

* Please **select up to 5 specific device attributes** that you are currently evaluating:

- ☐ Accessibility
- ☐ Adaptability
- ☐ Autonomy
- ☐ Benefit
- ☐ Comfort
- ☐ Compatibility
- ☐ Complexity
- ☐ Consistency
- ☐ Cost-effectiveness
- ☐ Customization
- ☐ Durability
- ☐ Ease of learning
- ☐ Ease of use
- ☐ Ergonomics
- ☐ Feasibility
- ☐ Functionality
- ☐ Helpfulness
- ☐ Independence
- ☐ Intuitiveness
- ☐ Learnability
- ☐ Meet user needs
- ☐ Mental demand
- ☐ Performance
- ☐ Physical demand
- ☐ Practicality
- ☐ [illegible]

- [ ] Quality
- [ ] Reliability
- [ ] Robustness
- [ ] Safety
- [ ] Technical requirements
- [ ] Understandability
- [ ] User-friendliness
- [ ] Utility
- [ ] Wearability
- [ ] Other
- [ ] Did not evaluate anything

QuestionPro

* Please specify what general usability evaluation methods you used to assess the chosen device attributes **with direct/indirect involvement of your users**?

The methods listed correspond to ISO/TR 16982:2002 = Ergonomics of human-system interaction — Usability methods supporting human-centred design

?

Accessibility

☐
☐
☐
☐
☐
☐
☐
☐

Adaptability

Autonomy

Benefit

Comfort

Compatibility

Complexity

Consistency

Cost-effectiveness

Customization

Durability

Ease of learning

Ease of use

Ergonomics

Feasibility

Functionality

Helpfulness

Independence

Intuitiveness

Learnability

Meet user needs

Mental demand

Performance

Physical demand

Practicality

Quality

Reliability

Robustness

Safety

Technical requirements

Understandability

User-friendliness

Utility

Wearability

Other

Did not evaluate anything

Please specify which of the following **performance-related measurements** you used:

Feel free to add OTHER if you do not find the measure(s) you used

- [ ] Time for task
  e.g. donning/doffing
- [ ] Task success/failure
  e.g. execution of activity of daily living
- [ ] Standardized functional test/measure
- [ ] Physiological measures
  e.g. Electromyography (EMG), Electrocardiography (ECG), metabolics
- [ ] Kinetic and/or kinematic analysis
- [ ] Other

Please specify

QuestionPro

Please specify which of the following **questionnaires and/or surveys** you used:

Feel free to add OTHER if you do not find the measure(s) you used

- [ ] After Scenario Questionnaire (ASQ)
- [ ] Assistive Technology Device Predisposition Assessment (ATD PA)
- [ ] Canadian Occupational Performance Measure (COPM)
- [ ] Intrinsic Motivation Inventory (IMI)
- [ ] Likert Scale - <u>Custom/self-made</u> (LS)
- [ ] NASA Task Load Index - <u>Original: weighted</u> (NASA TLX)
- [ ] NASA Task Load Index - <u>Raw: unweighted</u> (NASA RTLX)
- [ ] Nielsen's Attributes of Usability (NAU)
- [ ] Numeric Rating Scales - <u>Custom/self-made</u> (NRS)
- [ ] Open text questions -<u>Custom/self-made</u>
- [ ] Perceived Usefulness, Perceived Ease of Use (PUEU)
- [ ] Post-Study Usability Questionnaire (PSSUQ)
- [ ] Psychosocial Impact of Assistive Device (PIADS)
- [ ] Psychometric Scale to Assess the Satisfaction of Users with Assistive Technology (PYTHEIA)
- [ ] Purdue Usability Testing Questionnaire (PUTQ)
- [ ] The Questionnaire for User interaction Satisfaction (QUIS)
- [ ] Quebec User Evaluation of Satisfaction with Assistive Technology (QUEST 2.0)
- [ ] Rapid Assessment of Product Usability & Universal Design (RAPUUD)
- [ ] Summated Usability Metric (SUM)
- [ ] System Usability Scale - <u>Original</u> (SUS)
- [ ] System Usability Scale - <u>Adapted/customized/modified</u> (mSUS)
- [ ] Telehealthcare Satisfaction Questionnaire – Wearable Technology (TSQ-WT)
- [ ] Usability Metric for User Experience (UMUX & UMUX LITE)
- [ ] Usability Scale for Assistive Technology (USAT)

- [ ] Usefulness, Satisfaction, Ease of Use Questionnaire (USEQ)
- [ ] Visual Analogue Scales - Custom/self-made (VAS)
- [ ] Other
- [ ] Did not use any

* Please state your level of agreement with the following statements about **usability evaluation:**

	Strongly Disagree	Disagree	Neutral	Agree	Strongly Agree
I prefer quantitative over qualitative data	○	○	○	○	○
I prefer custom-made measures over standardized tools	○	○	○	○	○
I am confident we evaluated the usability of the device thoroughly	○	○	○	○	○
It was easy to find standardized measures for my context of use	○	○	○	○	○
I was able to compare my evaluation data with state-of-the-art benchmarks	○	○	○	○	○
The usability evaluation data improved the device development	○	○	○	○	○

What do you consider to be the **hardest** usability attribute to evaluate?

What tools would/did you use to assess it?

QuestionPro

What do you consider to be the **easiest** usability attribute to evaluate?

What tools would/did you use to assess it?
