# Supplementary material for: An analysis of usability evaluation practices and contexts of use in wearable robotics
Source: J Neuroeng Rehabil. 2021 Dec 9;18:170. doi: 10.1186/s12984-021-00963-8 (PMC8656061; doi:10.1186/s12984-021-00963-8)
Supplement: Supplementary file 2 — Additional file 2. Extension of Table 2 with all usability attributes. [file 12984_2021_963_MOESM2_ESM.pdf]

Additional file 2

Extension of Table 2 with all usability attributes

**Attribute**	**Times selected (100%)**	**Performance-related measurements**	**Questionnaire, Survey**	**Interview, unstructured oral feedback**	**Thinking aloud**	**Observation of users**	**Document-based methods**	**Model- or simulation-based approach**	**(Usability) Expert evaluation**
Functionality	47	31	17	15	7	18	4	10	10
Ease of use	46	18	27	23	12	20	7	5	11
Performance	40	32	12	7	6	15	5	12	5
Safety	40	14	11	11	8	17	9	8	9
Comfort	37	8	24	17	4	10	3	2	7
Benefit	26	18	15	9	4	9	3	4	4
Reliability	25	18	5	5	2	6	8	7	7
Ergonomics	23	4	9	5	3	12	7	3	5
Technical requirements	22	15	1	3	2	7	6	12	8
Wearability	22	7	9	9	5	12	8	5	7
Adaptability	21	4	6	11	7	9	3	3	3
Meet user needs	20	4	14	11	3	6	3	2	7
Autonomy	16	10	5	5	5	4	2	4	3
Feasibility	16	9	4	5	2	5	2	3	1
Intuitiveness	16	5	8	11	4	4	2	1	3
Robustness	15	7	4	3	2	5	2	6	3
User-friendliness	15	1	10	11	4	9	1	0	5
Cost-effectiveness	13	5	3	2	1	1	3	1	4
Ease of learning	13	7	5	5	2	2	2	2	1
Independence	12	4	7	5	1	5	0	2	2
Physical demand	11	4	5	2	1	4	0	2	1
Accessibility	9	2	2	3	3	2	1	1	2
Customization	9	4	1	3	1	4	2	2	1
Durability	9	2	2	2	2	4	2	2	2
Helpfulness	9	2	3	3	1	1	0	0	0
Practicality	8	1	4	4	0	5	0	0	3
Utility	7	6	5	4	3	3	1	0	2
Compatibility	6	4	1	2	0	1	2	2	2
Complexity	6	0	2	1	2	0	0	1	0
Consistency	6	2	2	1	0	2	0	3	1
Quality	6	4	1	1	0	3	2	0	4
Learnability	5	1	1	2	1	2	1	0	0
Mental demand	4	0	3	0	0	1	0	0	0
Understandability	3	1	1	1	1	1	0	0	1
**Total**		**254**	**229**	**202**	**99**	**209**	**91**	**105**	**124**
