# Supplementary material for: An analysis of usability evaluation practices and contexts of use in wearable robotics
Source: J Neuroeng Rehabil. 2021 Dec 9;18:170. doi: 10.1186/s12984-021-00963-8 (PMC8656061; doi:10.1186/s12984-021-00963-8)
Supplement: Supplementary file 3 — Additional file 3. Figure S1 and Tables S1 and S2. [file 12984_2021_963_MOESM3_ESM.pdf]

Additional file 3

As Figure S1 shows, the analyses of the other groupings further confirm the dominance of effectiveness as predominantly assessed dimension of usability when compared to efficiency and satisfaction. The mean efforts allocated for effectiveness are similar across groups and subgroups. In Table S1 and S2, we listed all p-values of the corresponding paired t-test (within subgroups, vertical, intra-subject) as well as the two-sample t-tests (across subgroups, horizontal, inter-subject). Regarding the Reviewers question, we can conclude that as shown in Figure S1, there was no significant difference found in the dimension-specific allocations between Form of Supervision subgroups. Still, a non-significant trend can that be observed is that the focus on satisfaction appears to increase, with decreasing level of supervision (from supervised, to aided, to unsupervised). The Technology Readiness Phases (TRP) grouping is the only analysis that also shows significant differences in the inter-subject comparisons (horizontal comparison).

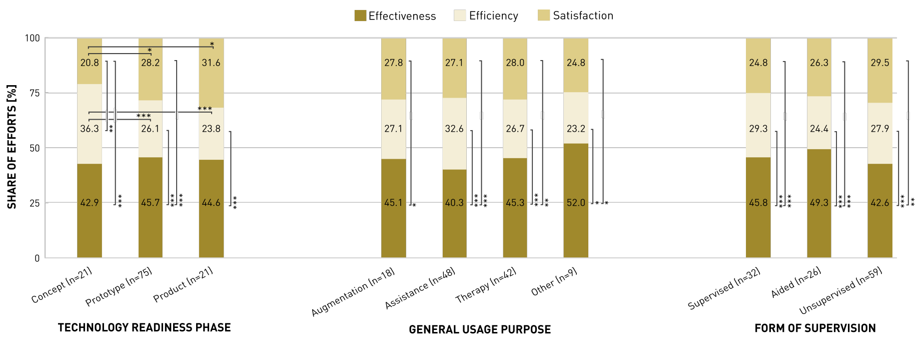

Figure S1: Evaluation effort allocation analysis of additional groups: From left to right, Technology Readiness Phases (TRP), General Usage purpose (GUP), Form of Supervision (FOS). * = p <0.05, ** = p <0.01, *** = p <0.001..

Table S1: Paired t-test p-values

	N	eff-efi	eff-sat	efi-sat
**TRP**				
concept (con)	21	0.3076023	0.0006965 ***	0.0047224 **
prototype (prt)	75	0.0000000 ***	0.0000017 ***	0.4685511
product (pro)	21	0.0005387 ***	0.1050373	0.1568491
**FOS**				
supervised (sup)	32	0.0009432 ***	0.0000320 ***	0.2909323
aided (aid)	26	0.0003697 ***	0.0003926 ***	0.7009228
unsupervised (uns)	59	0.0000380 ***	0.0025242 **	0.6533606
**GUP**				
Augmentation (aug)	18	0.2342268	0.0188284 *	0.4650196
Assistance (ass)	48	0.0000118 ***	0.0001181 ***	0.8459567
Therapy (the)	42	0.0000706 ***	0.0014425 **	0.7699517
Other (oth)	9	0.0208066 *	0.0367455 *	0.8585952

* = $p < 0.05$, ** = $p < 0.01$, *** = $p < 0.001$

Table S2: Two-sample t-test p-values

	Effectiveness (eff)	Efficiency (efi)	Satisfaction (sat)
**TRP**			
con-prt	0.502	0.005 **	0.050 *
con-pro	0.750	0.004 **	0.034 *
prt-pro	0.803	0.482	0.401
**FOS**			
sup-aid	0.415	0.218	0.702
sup-uns	0.372	0.639	0.201
aid-uns	0.104	0.300	0.420
**GUP**			
aug-ass	0.269	0.173	0.878
aug-the	0.293	0.185	0.862
aug-oth	0.068	0.191	0.721
ass-the	0.960	0.892	0.953
ass-oth	0.268	0.415	0.590
the-oth	0.323	0.509	0.626

* = $p < 0.05$, ** = $p < 0.01$, *** = $p < 0.001$
